# Adsorption of Pb(II) and Cr(VI) from Aqueous Solution by Synthetic Allophane Suspension: Isotherm, Kinetics, and Mechanisms

**DOI:** 10.3390/toxics10060291

**Published:** 2022-05-27

**Authors:** Yan Xia, Yang Li, Ying Xu

**Affiliations:** 1 School of Ecology and Environment, Zhengzhou University, Zhengzhou 450001, China; yan.xia@decarbon-mc.com; 2 School of Agriculture and Environment, Massey University, Private Bag 11222, Palmerston North 4442, New Zealand; 3 College of Resources, Sichuan Agricultural University, Chengdu 611130, China

**Keywords:** synthetic allophane, adsorption mechanism, selective absorption, heavy metal pollution

## Abstract

The adsorption of heavy metals on allophane has been extensively studied due to the properties of allophane special. However, the difference in adsorption behaviors and mechanisms of a metal cation and metal anion on allophane remains uncertain. The present study aimed to investigate the removal of Pb(II) and Cr(VI) onto synthetic allophane under variable pH, initial Pb(II) and Cr(VI) concentrations, and contact time. The results showed that the maximum adsorption capacity of allophane for Pb(II) and Cr(VI) was 88 and 8 mg/g, respectively. Equilibrium adsorption for Pb(II) was achieved in <2 min, but it took >12 h for Cr(VI). The response to changes in pH indicated the occurrence of electrostatic adsorption occurred during Cr(VI) absorption. XPS analysis suggested that reactions between predominant surface functional groups of allophane (Al-O- and Si-O-) and Pb(II) occurred through the formation of P-O bonds. The uptake mechanism of Pb(II) was based on a chemical reaction rather than a physical adsorption process. Synthetic allophane holds great potential to effectively remove aqueous metal ions for special wastewater treatment applications.

## 1. Introduction

The world is hurtling toward a future with dwindling water resources, and water pollution is one of the most compelling global concerns [1,2,3,4]. Heavy-metal pollution in water, as one of the most common sources of water pollution, has become a serious environmental issue caused by anthropogenic activities and natural processes [5]. For example, lead (Pb) is generally released into the environment through metal mining industries of acid lead batteries, paper, glass, and polishing industries, which may cause anemia, neurological dysfunction, and kidney damage, and chromium (Cr) causes diseases such as liver damage, nephritis, and stomach distresses and is also the major cause of nasal mucous ulcer [6]. To reduce the heavy-metal pollution in water, numerous methods have been developed including removal through adsorption, phytoremediation, bioremediation, chemical precipitation, coagulation, membrane filtration, ion exchange, and redox processes [7,8,9]. Among them, adsorption has been deemed as a promising alternative for metal pollution due to its easy obtainment, simple production, and operation with relatively high efficiency and low cost [10]. In previous studies, various adsorbents (e.g., biochar, carbon nanotubes, activated carbon, and minerals) have been used to remove heavy metals [11]. Finding a cost-effective and highly efficient adsorbent is still necessary.

One such adsorbent that has not been sufficiently investigated is allophane [12]. Allophane (1-2SiO_2_·Al_2_O_3_·nH_2_O) is a short-range ordered clay mineral of ubiquitous occurrence in soils of volcanic origin, which has abundant surface functional groups (e.g., Al-O, Si-O, and Si-OH) and a large specific surface area (>300 m^2^/g) [13]. Natural allophane spherule consisted of an outer diameter of ~5 nm, a wall thickness of 0.7–1.0 nm, and a perforation of ~0.3 nm [14]. The functional groups (e.g., Al(OH)_3_, (OH)Si(AlO)_3_, and (HO)Al(OH_2_)) were exposed to outer, wall, and perforation, respectively [15]. The hollow nanosphere structure of allophane is fundamentally composed of an outer layer of a gibbsite-like sheet with SiO_4_ tetrahedral attached to its interior with defects or perforations in the wall structure with diameters of around 0.3 nm [16]. This layered structure offers abundant sites for the absorption processes. Conceptually, given the application, the uniform shape, size, and inherent acidity of synthetic allophane are considered better than the natural ones [17]. However, the sorption properties of synthetic allophane are barely utilized in the remediation of wastewater. Basic studies are needed to explore the qualitative and quantitative assessment of the metal ion removal efficiencies and the determination of adsorption capacities of critical metal ions by using synthetic allophanes for water clean-up [18].

The present aim of this paper is to examine and compare the co-precipitation and adsorption characteristics of different metal ions to reveal the adsorption mechanisms of the allophane concerning heavy metal cations and anions. Here, Pb and Cr are regarded as representatives of common metal cations and anions, as they were found mainly in cations (Pb^2+^) and anions (CrO_4_^2−^) formed in the environment, respectively [19]. In this context, this study investigated the uptake of Pb(II) and Cr(VI) by synthesizing allophane at different conditions to figure out the adsorption mechanisms of the synthesized allophane. Specifically, synthetic allophane was used to remove Pb(II) and Cr(VI) in aqueous solution with varying pH, initial concentrations, and contact time. The results show that the maximum adsorption capacity of allophane for Pb(II) and Cr(VI) was 88 and 8 mg/g, respectively. The adsorption of Pb(II) is relatively high compared to other adsorbents, but the adsorption level of Cr(VI) is common. Furthermore, the equilibrium adsorption time for Pb(II) was much faster than Cr(VI). Interestingly, the Pb-adsorbed-synthetic allophane cracked and settled when adjusting pH to 7 due to the chemical reaction. The potential use of the synthetic allophane in heavy metal water processing technologies was also discussed.

## 2. Materials and Methods

### 2.1. Synthesis

**Figure 1:**
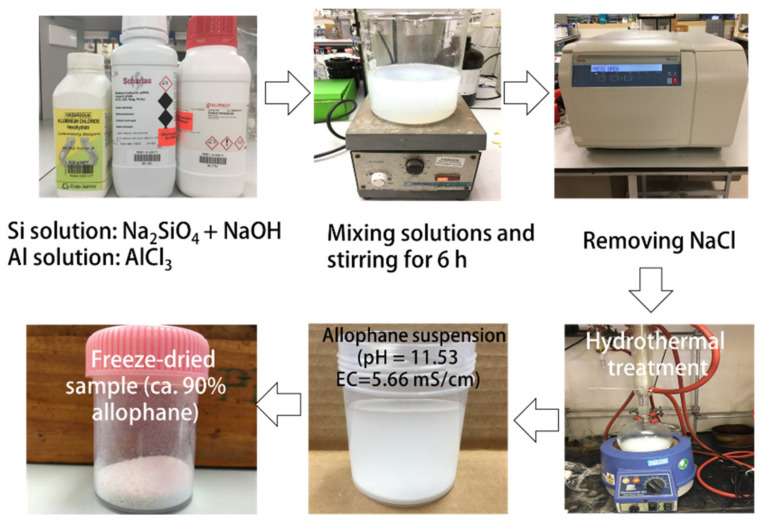
The scheme of the synthesis processes of allophane.

Allophane was synthesized based on the method reported by Ohashi et al. [16]. The fabrication processes are shown in Figure 1. Al source and Si source were AlCl_3_ and Na_2_SiO_4_, respectively. NaOH was added to Si solution for adjusting the alkalinity to favorable conditions for allophane synthesis. Both solutions were mixed rapidly with an atomic ratio of Al:Si of 4:3, then stirred for 1 hour at room temperature. After removing the by-product (NaCl) by centrifugation, the precursor was collected and hydro-thermalized at 95–100 ℃ for 48 h. Allophane was carried out through repeated washing with deionized water until pH is neutral. Allophane suspension was applied in this experiment.

### 2.2. Characterization of Adsorbent

Physiochemical properties of the synthesized allophane, including allophane content, morphology, and surface reactivity towards polar compounds and surface functional groups were analyzed [7]. The content of allophane was determined by acid oxalate extraction and sodium pyrophosphate extraction methods [20]. The crystallinity of synthetic allophane was determined by the X-ray diffraction (XRD) patterns, recording on a Bruker D8 Advance X-ray diffractometer using Cu Kα radiation. The morphology of allophane was shown by transmission electron microscopy (TEM, JEM-2100F, JEOL Ltd., Tokyo, Japan) analysis. Surface reactivity towards polar compounds of synthetic allophane was determined by water content of air-dried samples [21]. Surface functional groups of allophane were conducted by Fourier-transform infrared spectroscopy (Nicolet 5700, FTIR spectrometer). The chemical information of the materials was analyzed by X-ray photoelectron spectroscopy (XPS, K-Alpha, Al Ka radiation). Allophane suspension at a 1:500 (*w/v*) ratio was prepared, and the pH was adjusted by adding HCl and NaOH. It was used to measure the zeta potential of allophane in the pH range from 2 to 13 by Malvern Zetasizer Nano ZS. 

### 2.3. Adsorption Experiments

The stock solutions of CrO_4_^2−^ and Pb^2+^ with a concentration of 2000 mg/L were prepared using K_2_CrO_4_ and PbCl_2_. These solutions were further diluted for the desired concentrations of Pb and Cr. Adsorption experiments were carried out in 50 mL centrifuge tubes, where allophane suspension and the solution containing either Pb or Cr at the concentrations under study were added at the ratio of 1:500 (*w/v*) [22]. Adsorption parameters considered included initial concentration of Pb and Cr, contact time, and solution pH (adjusting by adding either 1 M NaOH or 1 M HCl), which were varied from 5 to 1200 mg/L, 0 to 24 h, and 2 to 10, respectively. After adsorption, suspensions were filtered by using 0.45 µm membrane, and the concentration of residual Pb and Cr in the filtrate was measured by using a microwave plasma-atomic emission spectrometer (4200 MP-AES, Agilent, Santa Clara, CA, USA), setting 405 nm for Pb and 425 nm for Cr. All experiments were conducted in three replicates. The results obtained were calculated and fitted as described below.

The adsorbed heavy metal ions onto synthetic allophane and removal percentage (%) were calculated by Equations (1) and (2), respectively.

(1) $$q_{e}=\frac{\left(C_{0}-C_{e}\right)}{m}\times V\;$$

(2) $$\%removal=\frac{\left(C_{0}-C_{e}\right)}{C_{0}}\times 100\;$$

where $q_{e}$ is the concentration of adsorbed heavy metal ions (mg/g), $C_{0}$ and $C_{e}$ are the initial and equilibrium concentration of heavy metal ions in solution (mg/L), m is the dray mass of adsorbent (mg), and V is the volume of the adsorbent solution (mL).

The Langmuir and Freundlich models employed as empirical isotherm models were expressed as Equations (3) and (4), respectively.

(3) $$q_{e}=\frac{Q_{m}K_{l}C_{e}}{1+K_{l}C_{e}}\;\;\;$$

(4) $$q_{e}=K_{f}C_{e}^{1/n}$$

where $q_{e}$ and $C_{e}$ were the same in Equation (1), $Q_{m}$ is the maximum adsorption capacity of synthetic allophane (mg/g), $K_{l}$ is the empirical affinity Langmuir coefficient (L/mg), $K_{f}$ is the Freundlich adsorption affinity coefficient (L/kg), and n is the Freundlich linearity constant, depending on the character of the adsorbent.

Non-linear isotherms were assumed as Langmuir models, as the shape can be expressed by a dimensionless constant separation factor or equilibrium parameter ($R_{L}$), and *R_L_* is defined as Equation (5) [23].

(5) $$R_{L}=\frac{1}{1+K_{l}C_{0}}$$

where $K_{l}$ (L/mg) is Langmuir model constant and $C_{0}$ (mg/L) is the largest initial concentration of metal solutions. $R_{L}>1,\;R_{L}=1,\;0<R_{L}<1,\;R_{L}=0$ indicate unfavorable, linear, favorable, and irreversible adsorption, respectively.

The pseudo-first-order and pseudo-second-order were employed to study the adsorption kinetics and expressed as Equations (6) and (7), respectively.

(6) $$q_{t}=q_{e}\left(1-e^{-k_{1}t}\right)\;\;$$

(7) $$q_{t}=\frac{t}{\frac{1}{k_{2}q_{e}^{2}}+\frac{t}{q_{e}}}\;\;\;$$

where $q_{e}$ and $q_{t}$ were the concentration of adsorbed heavy metal ion at equilibrium and at time t, respectively (mg/g), t was shaking time (h), and $k_{1}$ and $k_{2}$ were adsorption rate of the first order and second order, respectively.

## 3. Results and Discussion

### 3.1. Characterizations of Synthetic Allophane

**Figure 2:**
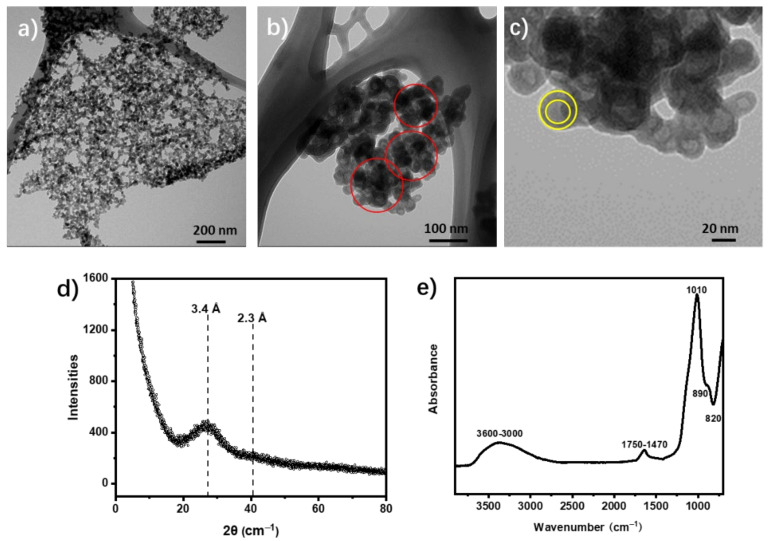
Transmission electron microscopy (TEM) images of synthetic allophane showing spherical (**a,b**) and hollow (**c**) morphology of allophane. PXRD pattern (**d**) and FTIR spectra (**e**) for synthetic allophane.

In this study, the synthetic allophane yield was ca. 90% of total product with the Al/Si ratio of 0.77. TEM images of synthetic allophane were shown in Figure 2, which shows the hollow structure of allophane clearly. Specifically, allophane spherules coalesced to form nanoaggregates with the diameter of ca. 100 nm. Consequently, those nanoaggregates can form larger allophane aggregates with the diameter of hundreds of nanometer (Figure 2a,b). The single synthetic allophane appeared as a nano-spherule with an external diameter of ca. 20 nm and a shell of about 2 nm (Figure 2c). Additionally, there were many pores within aggregates resulting in porous structure of allophane aggregates (Figure 2b), which is also beneficial for the absorption. As shown in Figure 2d, the XRD patterns of synthetic allophane exhibited two broad peaks at ~3.4 Å and ~2.3 Å, which are typical peaks for short-range ordered aluminosilicates such as allophane [24]. The specific surface area (SSAs) of synthetic allophane was tested to be a high level of 354 m^2^/g, which is related to its core–shell structure and aggregation state. The FTIR spectra of synthetic allophane is shown in Figure 2e. Identifiable FTIR features for synthetic allophane correspond to OH stretching (at 3600–3000 cm^−1^), organic complex of organic impurities (at 1750–1470 cm^−1^), Si-O-(Al) or Si-O-(Si) bonding (at 1010 cm^−1^), Si-OH bonding (at 890 cm^−1^), and Al-O and Si-OH bonding (at 800–400 cm^−1^) [17,25].

### 3.2. Adsorption Test

To investigate the absorption capacity of the synthetic allophane, Pb(II) and Cr(VI) as two typical heavy metal pollutants are used as targets. Different aqueous solutions with varying pH, initial concentrations, contact time, and electrolytes were tested.

#### 3.2.1. Effect of Concentration

**Figure 3:**
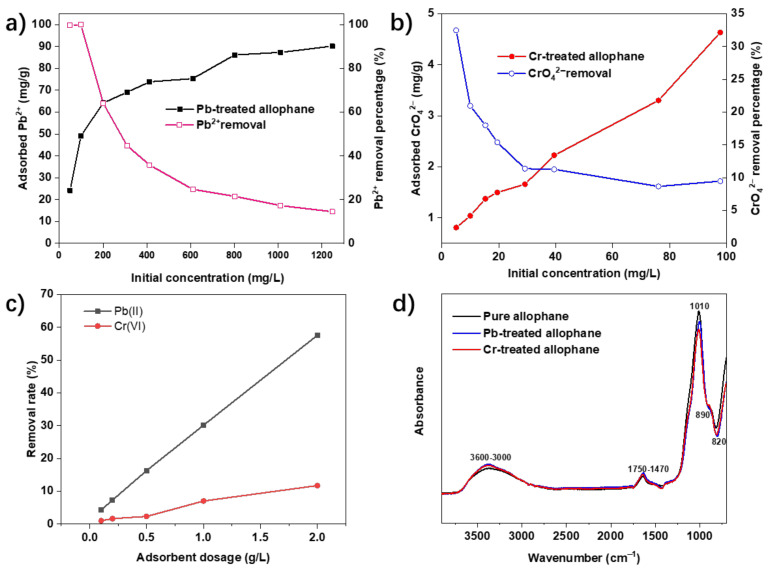
Effects of initial concentration of Pb^2+^ and CrO_4_^2−^ onto synthetic allophane: (**a**) Contact time of 24 h, pH at 5; (**b**) Contact time of 24 h, pH at 2. (**c**) Effect of adsorbent concentration onto synthetic with an initial concentration of Pb(II) and Cr(VI): 200 mg/L and 10 mg/L, respectively; Pb(II) solution pH at 5, Cr(VI) solution pH at 2; and (**d**) FTIR spectra for synthetic allophanes before and after absorption.

The initial concentrations of the Pb^2+^ and CrO_4_^2−^ solution had an obvious influence on the corresponding adsorptions. The dosage of the absorbent was 40 mg/L. The effect of initial concentrations of CrO_4_^2−^ and Pb^2+^ on the efficiency of adsorption is shown in Figure 3a,b. The Pb^2+^ adsorption capacities went up with increasing initial concentrations from 50 to 1200 mg/L. The increase in adsorption of Pb^2+^ was not significant as the initial concentration was higher than 800 mg/L, which was attributed to the increased competitive adsorption, for Pb^2+^ adsorption may be due to limited surface active sites of the adsorbent at high initial concentrations [26]. The maximum adsorption capacity reached 90.17 mg/g with a removal rate of 14.6%. The CrO_4_^2−^ adsorption capacities went up with increasing concentrations from 5 to 100 mg/L. The maximum adsorption capacity was 4.63 mg/g (removal percentage = 9.5%). The effect of adsorbent concentration of pollutions with a contact time of 24 h is shown in Figure 3c; the removal rate of the Pb(II) and Cr(VI) showed almost linear growth with the increase in absorbent dosage. The positive relationships between adsorbent dosage and removal rate of both Pb^2+^ and CrO_4_^2−^ could be explained by the adsorption site increasing according to the amount of adsorbent injected. [27]. Figure 3d shows the FTIR features for synthetic allophane after the adsorption, which indicates that the synthetic allophane is rarely changed in the chemical structure.

#### 3.2.2. Effect of Contact Time

**Figure 4:**
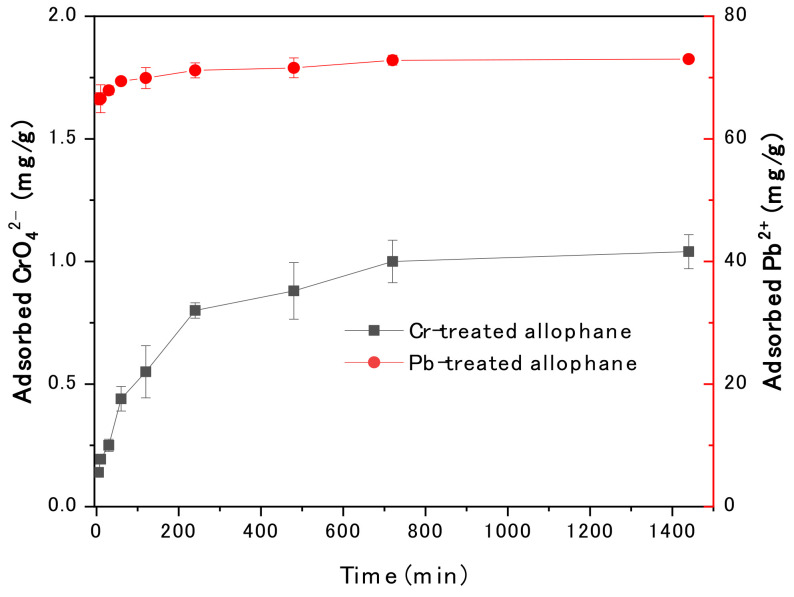
Effects of contact time on the adsorption of Pb^2+^ and CrO_4_^2−^ onto synthetic allophane (initial concentration of CrO_4_^2−^ and Pb^2+^ were maintained at 10 and 200 mg/L, respectively; pH of CrO_4_^2−^ and Pb^2+^ were maintained at 2 and 5, respectively; the dosage of the absorbent was 40 mg/L).

The effect of contact time of CrO_4_^2−^ and Pb^2+^ on the efficiency of adsorption is shown in Figure 4. Similarly, adsorption rate was high for both Pb^2+^ and CrO_4_^2−^ during the initial contact time (10 mins), and then adsorption rate gradually decreased. During the initial contact time, abundant vacant adsorption sites of allophane were provided to react with CrO_4_^2−^ and Pb^2+^, resulting in the high adsorption rate. However, by increasing the contact time, the available sites were gradually reduced due to the increased occupation of the sites by adsorbed CrO_4_^2−^ and Pb^2+^. Notably, the ca. 91% of the equilibrium adsorption capacity for Pb^2+^ was achieved within 2 minutes, and then the adsorption capacity gradually slowed down and reached a rough equilibrium in 10 minutes. The equilibrium adsorption capacity for CrO_4_^2−^ was achieved after 12 h of shaking. The equilibrium adsorption was generally considered related to the progressive saturation of the surface active sites for the CrO_4_^2−^ and Pb^2+^ adsorption [26,28]. 

#### 3.2.3. Effect of pH and Zeta (ξ) Potential

**Figure 5:**
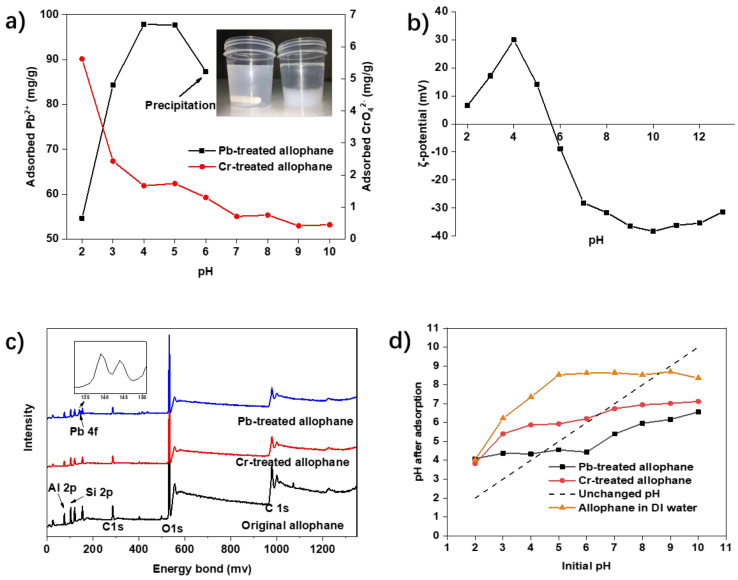
(**a**) Effect of pH on the adsorption of Pb^2+^ and CrO_4_^2−^ onto synthetic allophane (fixed shaking time was 24 h; initial concentrations of CrO_4_^2−^ and Pb^2+^ were 100 and 800 mg/L, respectively; dosage of the absorbent was 40 mg/L). (**b**) Zeta (ξ) potential of synthetic allophane. (**c**) XPS of the synthetic allophane. (**d**) pH changes in the solution of CrO_4_^2−^ and Pb^2+^ by applying synthetic allophane, data from the experiment of effect of pH.

As presented in Figure 5a, the pH of the solutions, ranging from 2 to 10, had different influences on the adsorption of Pb^2+^ and CrO_4_^2−^. Adsorption capacity for CrO_4_^2−^ gradually decreased with an increase in pH value, and would not further decrease when pH was higher than 9, which is probably because the hydrogen ions in solution assisted in adsorption. It is a typical electrostatic adsorption phenomenon of negative ions. In contrast, an obvious increase in Pb^2+^ adsorption ability (from 54.67 to 97.83 mg/g) was observed when pH increases from 2 to 4. The absorption decreased from pH at 5, and then could not be recorded when the pH value was higher than 6 due to precipitation. This phenomenon only occurs during the adsorption of Pb^2+^ ions, combined with the characteristics of short adsorption time, we suspect that certain chemical reactions occurred between Pb ions and the synthetic allophane. At any pH level, allophane plays better adsorption performance on Pb(II) than Cr(VI), which may also be related to their hydration energy and hydrated ionic radius [29]. During the adsorption process, hydrated water surrounded by metal ions would be dissociated and become free water, entropy-generated and spontaneous [30]. Additionally, lower hydration energy results in easier adsorption. Hydration energy is ranked as: Pb(II) < Cr(VI), and hydrated radius of Pb^2+^ and CrO_4_^2−^ are 0.401 nm and 0.375 nm, respectively [31]. As a result, Pb(II) was more likely to be adsorbed onto allophane due to its easy appearance as free ions.

To figure out the difference between the two absorptions, the surface charge of the synthetic allophane was measured. Its surface charge status at different pH is shown in Figure 5b. The zeta (ξ) potential value of the adsorbent increased from pH 2 to pH 4, then started to decrease to pH 10. This curve was a classical backward S shape, which indicated structural shifts at pH < 4 and pH > 10, or shielding effect due to excessive ions in the suspension at pH > 10. Point of zero charge/isoelectric point (PZC/IEP), where the zeta potential crosses positive and negative surface charge, was found at pH 5.6. In other words, the synthetic allophane surface was positively charged at pH < 5.6, and negatively charged at pH > 5.6. Due to the presence of aluminol groups, allophanes could either acquire or lose protons in response to pH changes, and were more protonated at lower pH [32]. Combined with the pH adsorption diagram, we can find that there is a correlation between the adsorption of the two metal ions and the zeta potential. When the pH is less than 5.6, the zeta potential is positive, and the adsorption efficiency of Pb^2+^ is continuously increased. Low adsorption capacity for Pb^2+^ was observed at low pH because surface sites were positively charged, resulting in electrostatic repulsion between Si-OH_2_^+^ and Pb^2+^. The reaction on the surface is shown as follows: Si-OH + H^+^ → Si-OH_2_^+^. The effect of electrostatic repulsion and negative charge decreased with raising pH. Pb has also been reported to have a larger ionic radius (1.20 Å), which has a lower charge density and is easily affected by the protonation of the surface groups, resulting in a reduction in the adsorption sites [33]. Additionally, the increased H^+^, having a great affinity for many complexation and cation exchange sites, would induce competition for the adsorption Pb^2+^ sites. A slight decrease in Pb^2+^ adsorption ability was also observed when pH was higher than 5, which may be attributed to the occurrence of PbOH^+^ as pH was higher than ca. 5.5 [34], while the absorption of CrO_4_^−^ was rarely affected by the change of the zeta potential. Then, the XPS was applied to test the elements and their conditions in the absorption. As shown in Figure 5c, there is no change in the overall structure and content of materials of the adsorbent before and after adsorption. The faint Cr peak was not found in the Cr-treated sample, which may relate to the small adsorption capacity on the one hand, and weak physical adsorption on the other hand. XPS spectra of the Pb level region was given in the inner part of Figure 5c; Pb–O bonding is involved, indicating peaks due to multiple oxidation states [35]. Active sites owned by allophane were Si-OH/Al-OH, -O-Si-O-/-O-Al-O-, and Al-O-/Si-O-. Adsorption occurs when the allophane-like oxygen ions on the surface of Pb-allophane bind to form a bond Si-O-Pb or Al-O-Pb. Pb, which acts as a Lewis acid, will receive a free electron pair of which acts as a base Lewis. The electron paired by O and Pb will form a coordinate covalent bond. 

In addition to the surface potential, pH changes of allophane suspension during adsorption were also recorded to determine H^+^/OH^−^ release. As shown in Figure 5d, at the initial lower pH, OH^−^ was released from allophane, then the H^+^ was released after the pH go to a high level. This phenomenon proves that the adsorbent itself has the function of a slow-release agent that releases anions and cations. With the addition of two salts, the pH of the solutions changed because PbCl_2_ presented acidic and K_2_CrO_4_ presented alkaline at the initial stage. The two intersections between dash line and metal solutions indicated that the solution pH did not change at initial pH 4.5 and 8.5 for Pb(II) solution Cr(VI) solution, respectively. When the initial pH was ranged from 2 to 6, the pH of Pb(II) solution after allophane application was stably at ca. 4, Similarly, when the initial pH was ranged from 7 to 10, the pH of Cr(VI) solution after allophane application was stably at ca. 8.5. However, the pH of the Pb-allophane solution suddenly loses its stability after the pH is over 6, which is due to the broken structure of the allophane. This implied there are at least two different adsorption mechanisms for both metals crossing the zeta potential. Combined with the zeta potential diagram, we can find that there is a correlation between the adsorption of the pH changing in the two solutions. The pH of the Cr-allophane solution equals 4 when the initial pH was 2, which means that the Zeta potential of the allophone is at the highest positive value. That is the reason why Cr adsorption from the initial pH equal to 2 is the highest amount of adsorption. The Cr absorption was almost stopped when initial pH was higher than 6; this is because the allophane became negative. This phenomenon also indicates that the adsorption of Cr is an electrostatic physical adsorption.

There is a conjecture about the breakage of Pb-allophane with pH adjustment after adsorption. As known, allophane generally dissolves to some extent in alkaline environments. At a lower pH (≤11.0), the preferential dissolution of polymerized silicates mainly occurred, but the amounts of dissolved Si and dissolved Al were small. The chemical composition and fundamental structure of the hollow spherules were barely affected [13]. The occupation of the O bonding after Pb absorption may reduce the stability of the original structure, which speeds up the disruption of the hollow spherules. This phenomenon is not necessarily a bad one in the application process. As known, only when the adsorbent is evenly dispersed in the solution can its adsorption effect improve. However, the nano-adsorbents with better dispersion should be separated from the solution by means of add magnetism or high-speed centrifugation [36]. Here, we can naturally separate the Pb-absorbed allophane from the solution by only adjusting the pH, which is easier to implement in the application process. The desorption experiments of the two ions were also conducted. After several treatments of desorption, the allophane disintegrated as shown in Figure 5a due to the instability of core–shell structure. Fortunately, the cost of mass production is low. Both harmless disposal and metal ion recovery can be achieved during disintegration and desorption.

### 3.3. Adsorption Behaviors

The adsorption isotherm and kinetics models were carried also carried out to provide a better understanding of the potential mechanisms behind adsorption behaviors on synthetic allophane.

#### 3.3.1. Adsorption Isotherm

**Figure 6:**
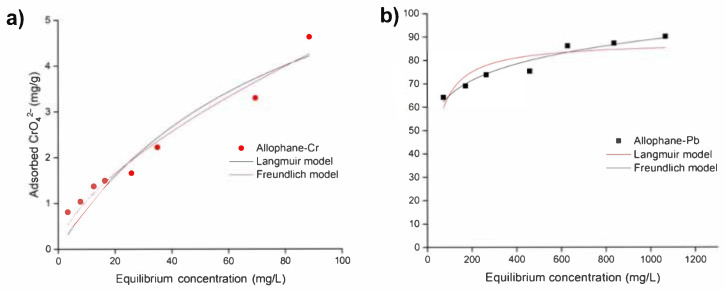
(**a**) Isotherm of CrO_4_^2−^ adsorption onto synthetic allophane. (**b**) Isotherm of Pb^2+^ adsorption onto synthetic allophane. The dosage of the absorbent was 40 mg/L.

The isotherm fitting results were shown in Figure 6 and Table 1. The regression coefficients (R^2^) of Langmuir and Freundlich models for CrO_4_^2−^ adsorption rates were 0.920 and 0.959, respectively, while for Pb^2+^ adsorption rates were 0.770 and 0.942, respectively. This result showed that the adsorption process of both CrO_4_^2−^ and Pb^2+^ onto synthetic allophane could be better described by the Freundlich model, indicating a heterogeneous adsorption process. Using Freundlich models, 1/n represented the exponent of non-linearity. It described the degree of curvature of fitting line and adsorption intensity. The value of n was 1.56 for CrO_4_^2−^ and 9.40 for Pb^2−^, resulting in a more curved isotherm for Pb^2+^ than the isotherm for CrO_4_^2−^. The values of $K_{f}$ for Pb^2+^ and CrO_4_^2−^ were 34.91 and 0.24, respectively. The isotherm type was constant partition, and the initial isotherm was linear, indicating constant partition. The great $K_{f}$ value indicated synthetic allophane had much greater adsorption capacity for Pb^2+^ than CrO_4_^2−^. The values of $R_{L}$ in Langmuir models were found to be 0.03 to 0.32 for the removal of Pb(II), and 0.53 to 0.967 for the removal of Cr(VI), respectively. This implied that the Pb(II) and Cr(VI) adsorption on allophane were both favorable adsorption processes.

#### 3.3.2. Adsorption Kinetics

**Figure 7:**
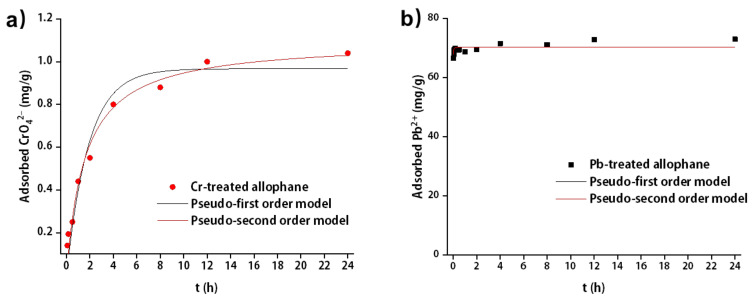
Kinetics of (**a**) CrO_4_^2^^−^ and (**b**) Pb^2+^ adsorption onto synthetic allophane. The dosage of the absorbent was 40 mg/L.

The kinetic fitting results for the absorption were shown in Figure 7 and Table 2. The pseudo-second order model could give a better simulation for both CrO_4_^2−^ and Pb^2+^ adsorption onto synthetic allophane due to higher R^2^. The value of R^2^ for Pb^2+^ adsorption in pseudo-second order model was 0.650 due to rapid absorption in a short period of time. The equilibrium adsorption capacities were similar to the experimental results that improved the reliability of pseudo-second order model. The adsorption rate constant $k_{2}$ for CrO_4_^2−^ and Pb^2+^ was 1.09 and 23.24 h^−1^, respectively, indicating that the adsorption of Pb^2+^ was about 21 times faster than CrO_4_^2−^.

### 3.4. Compare with Different Clay Materials

Many clay minerals (e.g., kaolinite, montmorillonite, and illite clays) have the ability to adsorb heavy metals. We compared the adsorption capacities of different adsorbents for Pb(II) and Cr(III/VI) and presented them in Table 3 and Table 4. Synthetic allophane possesses higher adsorption capacity onto Pb(II) than other clays. It is expected that the synthetic allophane have excellent potential for the removal of heavy metal cations (especially for Pb) without any modifications as they are low-cost and easily obtainable materials. However, its removal capacity for anions should be further improved.

## 4. Conclusions

The findings of the current study have offered an insight into the adsorption behaviors of heavy metal anion CrO_4_^2−^ and cation Pb^2+^ on the synthetic allophane and the mechanisms behind them. The results emphasize that the adsorptions were dominated by heterogonous surfaces, and the maximum adsorption capacity of Pb^2+^ was 10 times greater than that for metal anion CrO_4_^2−^. The ca. 91% of the equilibrium adsorption capacity for Pb^2+^ was achieved within 2 minutes, which is much faster than the equilibrium adsorption capacity for CrO_4_^2^^−^ (after 12 h of shaking). The adjustment of pH has an important effect on both adsorptions. As a result, the electrostatic attraction played important role in CrO_4_^2−^ adsorption, while a complex chemical reaction of Pb^2+^ adsorption occurred with the formation of P-O band. Further study of these mutual effects in the adsorption process on synthetic allophane is needed.

## Figures and Tables

**Table 1:** Isotherm parameters of CrO_4_^2−^ and Pb^2+^ onto synthetic allophane derived from Langmuir and Freundlich models.

Models	Parameters	Allophane-Cr	Allophane-Pb
Langmuir	$Q_{m}$ (mg/g)	8.09	88.08
	$K_{l}$ (L/mg)	0.01	0.03
	R^2^	0.920	0.770
Freundlich	$Q_{m}$ (mg/g)	0.24	34.91
	$K_{l}$ (L/mg)	1.56	9.40
	R^2^	0.959	0.942

**Table 2:** Kinetic parameters of CrO_4_^2−^ and Pb^2+^ onto synthetic allophane derived from pseudo-first order model and pseudo-second order model.

Models	Parameters	Allophane-Cr	Allophane-Pb
Pseudo-first order	$k_{1}$ (h^−1^)	0.53	70.37
	$q_{e}$ (mg/g)	0.97	174.34
	R^2^	0.94	0.34
Pseudo-second order	$k_{2}$ (h^−1^)	1.09	23.24
	$q_{e}$ (mg/g)	0.62	70.38
	R^2^	0.98	0.65

**Table 3:** Adsorption capacity of various clay materials towards Pb(II).

Adsorbent	Adsorption Capacity (mg/g)	References
Allophane	90.17	This study
Kaolinite	11.10	[37]
Turkish kaolinite clay	31.75	[38]
Modified kaolinite clay	32.20	[39]
Hal/alginate nanocomposite beads	325.00	[40]
Modified montmorillonite	131.58	[41]
Tunisian smectitic clay	25.00	[42]
Mt-chitosan composite	79.19	[43]
Natural beidellite clay	86.90	[44]
Beidellite	24.40	[44]
Turkish illitic clay	53.76	[45]
Hematite	16.34	[46]
Bentonite/thiourea-formaldehyde composite	14.38	[47]
Natural bentonite	85.47	[48]
Volcanic tuff	16.81	[49]
Silicon nanotube	42.85	[50]
Bentonite	28.00	[51]
Illite-smectite clay	131.23	[52]
Shanghai silty clay	26.46	[53]
Modified bentonite	123.30	[54]

**Table 4:** Adsorption capacity of various clay materials towards Cr(III/VI).

Adsorbent	Adsorption Capacity (mg/g)	References
Allophane	8.09	This study
Halloysite nanotube	6.90	[55]
Halloysite (m-HNTs/Fe3O4)	49.81	[56]
Raw Ca-montmorillonite	12.44	[57]
Humic acid modified Ca-montmorillonite	15.65	[57]
Modified montmorillonite	11.97	[58]
Modified montmorillonite	18.05	[59]
Dodecylamine modified Montmorillonite	23.69	[60]
Natural Akadama clay	4.29	[61]
Illite	0.27	[62]
Bentonite	4.68	[63]
Clay-perlite-iron	0.122	[64]
Illite-smectite clay	36.91	[52]
Shanghai silty clay	1.85	[53]
Sepiolite	27.05	[65]

## Data Availability

Not applicable.
